# Characterization of bacterial community changes and antibiotic resistance genes in lamb manure of different incidence

**DOI:** 10.1038/s41598-019-46604-y

**Published:** 2019-07-12

**Authors:** Ling-cong Kong, Bo Wang, Yi-ming Wang, Ren-ge Hu, AtiahLujia Atiewin, Duo Gao, Yun-hang Gao, Hong-xia Ma

**Affiliations:** 1 Department of Basic Veterinary Medicine, College of Animal Science and Technology, Jilin Agricultural University, Changchun, China; 2 Liaoning Testing & Inspection Center for Quality & Safety of Veterinary Drugs, Feed and Livestock Products, Liaoning, China; 3 The Key Laboratory of Animal Production, Product Quality and Security, Ministry of Education, Jilin Agricultural University, Changchun, China

**Keywords:** Bacterial pathogenesis, Pathogens

## Abstract

Bacterial enteritis is the most important disease in lamb for breeding greatly affects the growth of animals. Changes in the community of intestinal flora can cause the disorder of the colonic environment induced diarrhea. This study aimed to investigate the relationship between the incidence of bacterial enteritis and the number of intestinal microbiome, then the prevalence of drug-resistant genes was detected. Fecal samples were collected at five fattening sheep farms with different incidence of bacterial enteritis, pathogenic bacteria were isolated and identified, drug sensitivity tests were performed. Then, changes in number and structure of intestinal flora were compared by 16S rDNA V3-V4 region high-throughput sequencing, and the ARGs were detected using high-throughput real-time PCR. Our results revealed that the microbial communities were positively correlated with the incidence of bacterial enteritis in different farms. Bacterial communities were higher in YJ (with highest incidence of diarrhea) than any other farms. However, the ARGs seemed not to be more affected by the incidence of bacterial enteritis, but one of the significant findings to emerge from this study is that MCR-1 and NDM are detected in manure. This study has provided an insight of the changes occurring in intestinal flora and AGRs in fattening sheep farms with diverse incidence of bacterial enteritis.

## Introduction

Bacterial enteritis inlamb are a serious disorder affecting its weight gain resulting into economic losses, especially young sheep and goat are strongly affected by this condition leading to death due to malnutrition and dehydration, the costs associated with bacterial enteritis, including deaths, lost productivity and treatment, have been estimated at $10–29 million annually^1–3^. *Escherichia coli*, *Salmonella* and *Yersinia spp*. are the important bacterial agents associated with enteritis, but sporadic outbreaks associated with *Campylobacter* or *Salmonella spp*. have been reported^4^.

The intestinal microbiome has important effects on the host has revolutionized thinking about disease and health^5,6^. Changes in the community of intestinal flora can cause the disorder of the intestinal environment induced diarrhea Intestinal microbiome. Fecal microbiota transplantation (FMT) has been considered a highly effective treatment method for enteritis^7^ and has been used successfully to treat clostridium difficile infection (CDI) by the restoration of microbial populations. In animal production, recently some study has been able to explore the role of the intestinal microbiome in inflammatory bowel disease of broiler and pig, and there is growing evidence that FMT is an effective treatment for inflammatory bowel disease, but little guidance exists of sheep of FMT programs. Recently, diarrhoeal diseases have caused damage to meat sheep farming of Jilin provincs of China, but given the limitations of current diagnostic and therapeutic modalities, traditional drug therapy not only destroys intestinal flora, but also causes the spread of drug-resistant genes. The types of resistant genes in feces have been found in pig farms, chicken farms, and cattle farms. However, the type and abundance of resistant genes in faecal feces have not been reported. The objective of this study is to describe the relationship between the incidence of bacterial enteritis and the intestinal microbiome, then the prevalence of drug-resistant genes was detected, to help guide future FMT program implementation, and assess the risk of drug resistance.

## Materials and Methods

### Morbidity statistics and Sample collection

Bacterial enteritis was monitored in five fattening sheep farms (CC, BC, GZL, CL, and YJ) with different management levels and there are 500 sheep were monitored in each fattening sheep farm. These samples were collected from anus swabs of lamb diarrhoea. Pathogenic bacteria were isolated and identified using PCR and pathogenicity tests of mice as previously described^8^.

A susceptibility profile was established by broth microevolution, as recommended by the Clinical and Laboratory Standards Institute guidelines in VET01-A4 (CLSI 2013)^9^. The antimicrobial agents tested included enrofloxacin (ENR), florfenicol(FFC), cefotaxime (CEQ), tetracycline (TET), doxycycline (DOX), sulfamethoxazole/trimethoprim (SXT), gentamicin (GMS), tilmicosin (TIL). The breakpoints were adopted from CLSI document VET01-A4. The reference strain Escherichia coli ATCC 25922 served as quality control. In the end, the incidence of bacterial diarrhea in each farm was recorded.

Then, to study the relationship between the incidence of bacterial diarrhea of sheep and the intestinal microbiome. Samples were taken from freshly excreted feces at the selected farms. Fifty samples were collected at each farm. Every five samples were mixed. After freeze-drying, every five samples of the same farm were mixed and sieving them through a 2 mm meshes. The samples were stored at −80 °C for DNA extraction. All methods were carried out in accordance with the US NIH guidelines and protocols for the laboratory animal use and proper care, approved by the Animal Care and Use Committee of the Jilin Agricultural University.

Approved by the Animal Care and Use Committee of the Jilin Agricultural University.

### DNA extraction and purification

Total DNA was extracted using the PowerSoil DNA Kit (Anbiosci, Shenzhen, China). The DNA extraction was performed following the manufacturer’s protocol. The quality and concentration of the purified DNA were determined by NanoDrop ND-2000, and 1.5% agarose gel electrophoresis.

### Amplification of 16S rRNA gene sequences

The 16S rRNA V3 + V4 region was amplified by polymerase chain reaction (PCR). The primer was F: (5′-ACTCCTACGGGAGGCAGCA-3′) and R: 5′-GGACTACCGGGGTWTCTAAT-3′, which were provided by Biomarker Technologies (Beijing). The reaction was performed in a final volume of 25 µL containing DNA (10 ng), 4 µL 5 × FastPfu Buffer, 2 µL 2.5 mMdNTP, 1.0 U of Taq Polymerase,0.8 µL of each primer 5 µM, 0.2 µL BSA, and distilled water. The PCR cycling parameters were 95 °C for 3 min, reactions were run for 30 cycles of 95 °C for 30 s, 55 °C for 30 s, and 72 °C for 45 s, and a final extension at 72 °C for 10 min. The PCR products were separated on 1.0% agarose gel electrophoresis.

### Illuminapaired-end sequencing

The PCR products were sequenced and merged by Biomarker Technologies (Beijing, China) using Illumina paired-end protocoland Pandaseq^10^.

### Sequence analysis

The sequencesdata with 97% similarity were clustered into different Operational Taxonomic Units (OTUs)^11^. Then the data were analyzed through the Silva reference gene database (http://www.arb-silva.de/). The composition of each sample community was recorded. The alpha diversity index was evaluated by Mothur (V.1.11.0)^12^. To show the difference between samples, Principal Component Analysis (PCA) and principal coordinates analysis (PCoA) was performed to assess the bacterial composition of samples^13^. Then the highest abundance of bacteria and potential pathogens was screened and analysed.

### High throughput qPCR

The ARGs related to commonly used drugs were detected in the samples using high throughput real-time PCR (Wafergen Inc. USA). These included aminoglycoside resistance genes (*aac*C(6′)I1, *aac*C2, *aac*C4, *str*A, *str*B), Beta -Lactamase resistance genes (*bla*CTX-M, *bla*SHV and *bla*TEM), Tetracycline resistance genes (*tet*B, *tet*C, *tet*E, *tet*O, *tet*Q), Macrolide-Lincosamide-Streptogramin (MLSB) resistance genes (*mph*A, *mph*B, *erm*A, *erm*B, *erm*C). Sulfonamide resistance genes (*sul*I, *sul*II), quinolone resistance genes(*qnr*A, *qnr*S), Polymyxin resistance gene(MCR-I), New Delhi metallo-β-lactamase (NDM-I) and integrase gene (*int*-I). The primers list can be found as Supplementary Table S1. Ct values with value < 31 in more than two replicates were recorded, and the values of amplification efficiency <90% or >110% were discarded. Then the abundance of each gene was calculated as 2Ct, where Ctrepresents the difference in the Ctvalue for the 16 S rRNA gene minus that for the ARG^14–16^.

## Results

### Morbidity statistics and antibiotic resistance

From the 486 sampled in the study, a total of 187 pathogenic bacteria were obtained. Detailed information about the sampling sites is presented in Table 1. The incidence of bacterial diseases is lower in farms with high levels ofmanagement. The incidence was 0.01%, 0.02% and 0.5% in farms named CC, BC and GZL respectively. But the incidence of bacterial diseases is higher in farms with poor management levels. The incidence is 7% and 12% in farms named CL and YJ respectively.

**Table 1:** *In vitro* susceptibility of clinical isolatesoffive farms and morbidityofincidencerate.

Farms	Species	No. of isolates	incidence rate	No. of resistance isolates							
				ENR	FFC	CEQ	TET	DOX	SXT	GEN	TIL
CC	*Escherichia coli*	10	0.01%	3	1			6	1	1	1
BC	*Escherichia coli*	20	0.02%	2	1	1		4	2	3	1
GZL	*Escherichia coli*	5	0.5%	3	4	1	3	4	5	5	5
CL	*Escherichia coli*	67	7.0%	23	12	7	49	54	59	25	22
	*Aeromonastrota*	3		1	1	1	2	2	3	1	1
YJ	*Streptococcus*	17	12.0%	4	6	3	9	14	17	7	4
	*Escherichia coli*	56		21	16	5	25	41	51	40	32
	*clostridiumwelchii*	3		1			3	3	3		
	*Moraxellalacunata*	2		2			1	2	3	2	2
	*PlesiomonasShigeloides*	4		2	1		1	1	3	1	1

ENR, enrofloxacin; FFC, florfenicol; CEQ, cefotaxime; TET, tetracycline; DOX, doxycycline; SXT, sulfamethoxazole/trimethoprim. GMS, gentamicin; TIL, tilmicosin.

Importantly, the 187 pathogenic bacteria was tested for their susceptibility to eight antimicrobial agents using the AGAR dilution assay. Lots of isolates were resistant to ENR, TET, DOX, SXT, GEN and TIL. Lots of isolated were highly susceptible to FFC and CEQ (Table 1).

### Changes in bacterial community

**Figure 1:**
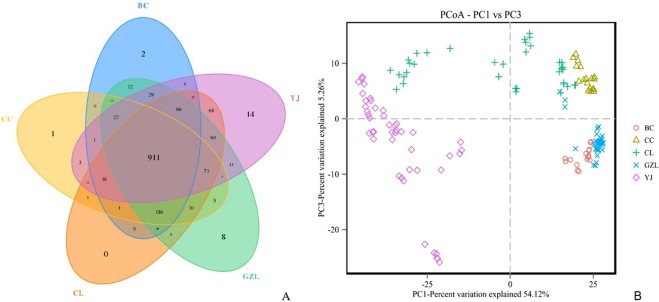
Differences in bacterial community diversity, richness and structures between different sheep farms. (**A**) Community diversity and richness between high and low weight feces (both hard and soft feces).

Our study investigated the bacterial community structure in five fattening sheep farms with a different incidence of bacterial enteritis. According to 16S rRNA genes Illumina sequencing data, a total of 12,004,981 reads were obtained from 150 samples (includes three biological replications), after filtration, 9,977,647 clean tags were generated, with an average of 66,518 clean tags per sample. Then, the total of 1592 predictive OTUs with 97% similarity was obtained among the five groups. As shown in Fig. 1A, the Venn diagram was used to measure the distribution of 1592 OTUs among the different samples. The 911 OTUs is common, but 14 specific OTUs were consulted on the YJ farm. So, there may be more atypical bacterial strains and a potential pathogen that could be isolated from the YJ farm.

The PCoA based on unweighted unifrac is shown in Fig. 1B. Samples from different groups were clustered separately. The samples at 0 m and 25 m could be distinctly separated from those at 0 m and −25 m by PC1 (54.12%), and samples at-10 m could be distinctly separated from the others by PC3 (5.26%). The results indicated that the bacterial communities were positively correlated with the incidence of different farms. Bacterial communities were similar between BC, CC and GZL, All sample lines were close to each other and had a low-slope, suggesting high species evenness in all samples. And through the bacterial diversity in CC samples was higher than that of other samples. But, bacterial communities were extremely diverse in YJ and CL samples.

**Figure 2:**
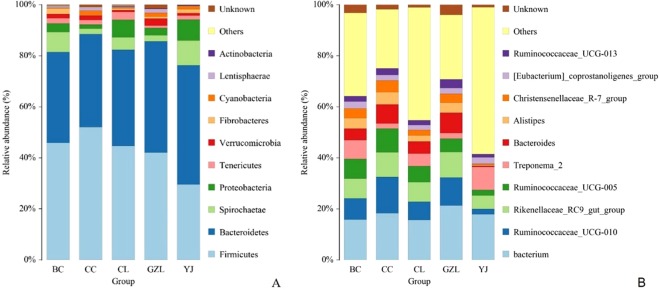
Proportion of taxonomical phyla in different sheep farms. BC: The incidence of bacterial disease was 0.02%; CC: The incidence of bacterial disease was 0.01%; CL: The incidence of bacterial disease was 7%; GZL: The incidence of bacterial disease was 0.5%; YJ: The incidence of bacterial disease was 12.

The relative abundance of the top 10 phyla is shown in Fig. 2A. *Firmicutes* and *Bacteroidetes* were the dominant phylum in all samples, reaching a proportion of 32.3–48.6%. *Acidobacteria* was the subdominant phylum with an abundance of 7.7–15.0%, followed by *Spirochactae* (4.2–15.0%) and *Proteobacteria* (1.8–9.6%). As the incidence of bacterial enteritis increases, the abundance of *Firmicutes* decreased, whereas that of *Bacteroidetes* increased. At the genus level, the relative abundance of the top 10 genera together only accounted for 7–22% of the total composition (Fig. 2B), total of 9 genus were found, and *Bacterium*, *Ruminococcaceae*_UCG-010, *Rikenellaceae*, *Ruminococc*_UCG-005, *Treponema*-2, *Bacteroides*, *Alistipes*, and *Christensenellaceae*_R-7_group were the dominant genus in all the samples. But, in the farm with a high incidence of bacterial enteritis, the abundance of the dominant genus is relatively lower than others.

To further estimate if samples were a relationship between the incidence of bacterial enteritis and the intestinal microbiome. KRONA was conducted at the genus level, the types and abundance of potential pathogens were detected. The results were shown in Table 2. 14 genus of potentially pathogenic bacteria were found, *Mycoplasma*, *salmonella*, *streptococcus* and other common clinical pathogenic bacteria were detected in all samples. As the incidence of bacterial enteritis increases, the variety of potentially pathogenic bacteria increased. Among, *mycoplasma*, *Actinomyces*, *Campylobacter*, *Fusiformis*, *Pasteurella*, *Klebsiella, Shigella*, *Salmonella*, *Streptococcus, Haemophilus*, *Mycobacterium* were found in Cl and YJ, what’s more, Brucella was found in YJ, and a worker in YJ has been infected with *Brucella* (Table 2).

**Table 2:** The types and of potential pathogens in all samples.

Potential pathogenic bacteria	Farms				
	YJ	CL	CC	BC	GZL
*Mycoplasma*	+	+	−	−	−
*Brucella*	+	−	−	−	−
*Actinomyces*	+	+	−	−	+
*Campylobacter*	+	+	+	+	+
*Fusiformis*	+	+	+	+	−
*Pasteurella*	+	+	−	−	+
*Klebsiella*	+	−	+	−	−
*Shigella*	+	−	+	+	−
*Salmonella*	+	+	−	+	−
*Streptococcus*	+	+	−	−	−
*Haemophilus*	+	+	−	−	−
*Mycobacterium*	−	+	−	−	−
*Rickettsia*	−	+	−	−	−
*Aeromonas*	−	−	+	−	−

### Changes in the abundance of ARGs

A total of ARGs were detected, although some were rare. Details of q-PCR assay validation results are listed in Figs S1 and S2 and the abundance of each drug resistance gene in each group can be found as Supplementary Table S2. The antibiotic resistance mechanisms of the ARGs include four main types: Antibiotic deactivation (42%), cellular protection (25%), efflux pump (11%), and transposon (5%). Overall the resistance genes for Tetracycline (21%), aminoglycoside (20%), MLSB (13%) and Beta-Lactamase (13%) were the most four dominant types in different incidence farms samples, and at the same time resistance genes for Sulfonamide (6%) and Quinolone (5%) were detected.

**Figure 3:**
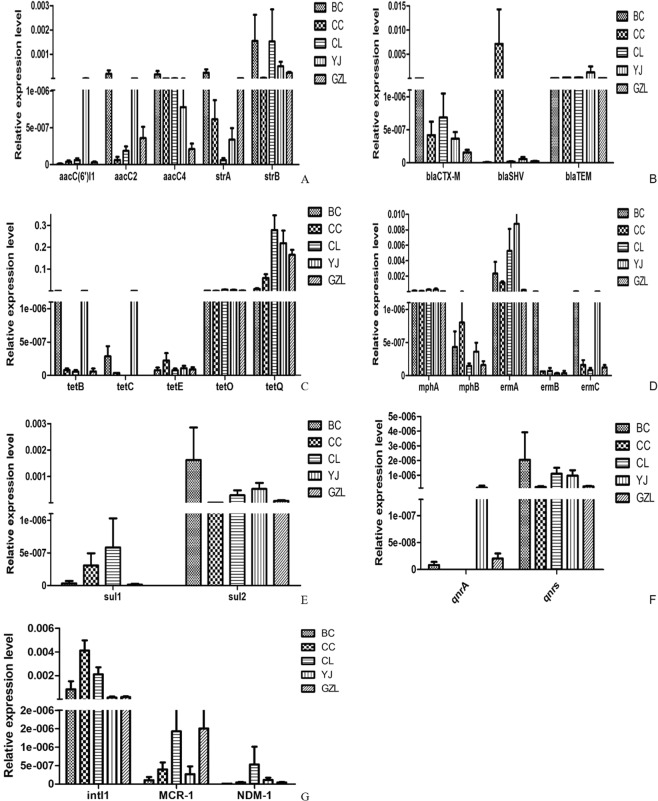
Relative abundance of resistance genes in different groups. (**A**) Relative abundance of Aminoglycoside resistance genes in different groups. (**B**) Relative abundance of Beta Lactamase resistance genes in different groups. (**C**) Relative abundance of Tetracycline resistance genes in different groups. (**D**) Relative abundance of MLSB resistance genes in different groups. (**E**) Relative abundance ofSulfonamide resistance genes in different groups. (**F**) Relative abundance of quinolone resistance genes in different groups. (**G**) Relative abundance ofintI1, MCR-1, NDM-1 in different groups.

As seen in Fig. 3, the total relative abundance of ARGs from samples ranged from 8.99 × 10^−9^ to 3 × 10^−1^. The relative abundance of tetracycline genes was higher than others, the relative abundance of *tet*O and *tet*Q was above 10^−3^. In the farms with a high incidence of bacterial disease, the abundance of the drug-resistance gene is higher, and There are more kinds than others. For example, the relative abundance of aminoglycoside resistance genes (*aac*A4, *str*B), beta -Lactamase resistance genes (*bla*TEM), tetracycline resistance genes (*tet*O, *tet*Q), MLSB resistance genes (*erm*A, *erm*C), sulfonamide resistance genes (*sul*II), quinolone resistance genes (*qnr*S) and integrase gene (*int*-I) were higher. But, Some resistance genes are also higher in the lower incidenceofbacterial disease, such as the relative abundance of *bla*SHV, *int*-1, *qnr*S, *sul*-2 and *erm*A are higher in CC and BC than others, especially, the relative abundance of *bla*SHVinCC are very highest, was above 5 × 10^−4^. More importantly, *mcr*-1 and NDM are also detected in manure. In particular, the abundance of *mcr*-1 and NDM-1 is over 10^−7^.

## Discussion

Lamb bacterial enteritis is an important disease that can cause economic loss and is one of the most common reported diseases^17^. Gut microorganism plays an important role in health and disease, for example, gut probiotics, can lead to the composition and specific changes of gastrointestinal microorganisms, thus conferring benefit(s) upon host health^18^. Describes the relationship between the incidence of bacterial enteritis and the intestinal microbiome is of significant economic importance. In the present study, 158 *E. coli*, 17 *Streptococcus*, 4 *PlesiomonasShigeloides*, 3 *Aeromonastrota*, 3 *clostridiumwelchii* and 2 *Moraxellalacunata* isolates were isolated from diarrheic samples. Our results revealed that the incidence of bacterial enteritis related to management level. The incidence of bacterial enteritis is higher in farms with poor management levels, for example, the incidence is 7% and 12% in farms named CL and YJ respectively. Among these pathogenic agents, E. coli is the most common and important ones. Higa toxin-producing *E. coli* (STEC) and enteropathogenic *E. coli* (EPEC) are frequently detected in small ruminants with diarrhea in further studies. Diarrhea associated with *E. coli* infections is often treated with antibiotics. However, therapy may be unsuccessful due to resistant isolates in animals^19^. Different patterns of antibiotic-resistant have been reported in sheep E. coli isolates^20^. In our study, all isolates were tested for their susceptibility to eight antimicrobial agents. Lots of isolates were resistant to ENR, TET, DOX, SXT, GEN, and TIL. Some of isolates were highly susceptible to FFC and CEQ. All isolates were found to be multidrug resistant.

Gut microbiota is recognized as the “second genome” playing a significant part in the animal body. Previously, many studies have studies that the gut microbial population interacts with the host immune system promoting the normal development and maturation of immune cells to produce cytokines thus influencing the host neurophysiology^21–23^. However, till now little is known about the characterization of bacterial community changes and antibiotic resistance genes in lamb with a different incidence of bacterial enteritis. In this study, we documented the composition and structure of intestinal microflora were significant differences in different farms, and it was positively correlated with the incidence. Among them, the two fields with a high incidence of CL (12% incidence) and YJ (7% incidence), the spatial distribution of samples was farther away from the field with lower incidence, and the distance between the samples within the group was larger, indicating the higher species richness of samples of these two groups. The distances between samples in the three groups with a relatively low incidence of CC (incidence rate 0.01%), BC (incidence rate 0.02%) and GZL (incidence rate 0.5%) were relatively small, and species diversity was also relatively small. At the phylum level, the abundance of *Streptomyces* and *Bacteroidetes* were the highest among the five groups, the structural differences of the top ten species were more obvious at the genus level, and the species with the highest abundance in the CC, BC, and GZL groups were *Rumenococcus*, and the species with the highest abundance in the CL and YJ groups were the *Alistipes*and the *Treponema*. As the incidence of bacterial enteritis increases, the abundance of *Firmicutes* decreased, whereas that of *Bacteroidetes* increased. Forthe ruminants, *Firmicutes* play a major role in degrading the fiber and cellulose^24^. Clinical pathogens such as *Mycoplasma*, *Salmonella*, and *Streptococcus* were identified in five regional samples of KRONA analysis, the composition of microorganisms were more complex in CL and YJ, and the pathogenic bacteria species were significantly more than CC, BC, and GZL groups.

The use of antibiotics can effectively kill pathogenic microorganisms, but at the same time, it also leads to the spread of antibiotic resistance genes (ARGs)^25^. Some ARGs have been shown to exist in transposons, integrons, or plasmids, which are mobilizable elements that can be transferred among bacteria The migration and transformation of ARGs in the enteric microorganism is potentially more harmful, it can cause pathogens to become resistant to antibiotics. In this study, antimicrobial resistance genes were studied in all samples. The antibiotic resistance mechanisms of the ARGs include four main types: Antibiotic deactivation (42%), cellular protection (25%), efflux pump (11%), and transposon (5%). Overall the resistance genes for Tetracycline (21%), aminoglycoside (20%), MLSB (13%) and Beta-Lactamase (13%) were the most four dominant types in different incidence farms samples, and at the same time resistance genes for Sulfonamide (6%) and Quinolone (5%) were detected. The detection rate of the BC group was 91.2%, while the detection rate of the remaining CC. GZL, CL, and YJ groups was 81.6%, 72%, 60% and 81.6%, respectively. Previous research has shown that the detection of tetracycline, sulfonamide antibiotic resistance genes and type 1 integrase genes in livestock and poultry feces is more serious^26^. In this study, the detection rate of the tetracycline resistance gene is the highest, may be related to the widespread use of tetracycline drugs in the veterinary clinic. And there is no correlation between the detection of resistance genes and farms with a different incidence of bacillary enteritis. The resistance genes of*str*A and *bla*SHV were detected more frequently in the field with a low incidence. It is possible that some drugs are used to prevent disease in farms with low incidence leading to the increased content of resistance genes in their feces. More importantly, *mcr*-1 and NDM are also detected in manure, the abundance of *mcr*-1 and NDM-1 are over 10-7. The emergence and spread of NDM-1-producing Enterobacteriaceae have resulted in worldwide public health risk, then, the emergence of MCR-1 heralds the breach of the last group of antibiotics. Some study has shown that *mcr*-1 is prevalent in hatcheries, but *bla*NDM quickly contaminates flocks through dogs, flies and wild birds^27^.

## Conclusion

This study reveals bacterial changes and the occurrence of ARGs in five fattening sheep farms with a different incidence of bacterial enteritis. Our results revealed that the microbial communities were positively correlated with the incidence of bacterial enteritis in different farms. However, the ARGs seemed not to be more affected by the incidence of bacterial enteritis, but more importantly, *mcr*-1 and NDM are detected in manure. This study has provided an insight into the changes occurring in intestinal flora and AGRs in fattening sheep farms with a diverse incidence of bacterial enteritis. However, more in-depth studies are needed to review potential factors that contribute to microbial changes and the mechanism through which ARGs occur and propagate.

## Supplementary information

Supplementary Information

## Data Availability

The datasets generated during and/or analyzed during the current study are available from the corresponding author on reasonable request.
