# Supplementary material for: Characterization of bacterial community changes and antibiotic resistance genes in lamb manure of different incidence
Source: Sci Rep. 2019 Jul 12;9:10101. doi: 10.1038/s41598-019-46604-y (PMC6625992; doi:10.1038/s41598-019-46604-y)
Supplement: Supplementary file 1 — Supplementary Information [file 41598_2019_46604_MOESM1_ESM.pdf]

Characterization of bacterial community changes and antibiotic resistance genes in lamb manure of different incidence

Ling-cong KONG[1¶], Bo WANG[1¶], Yi-ming WANG[1], Ren-ge HU[1], AtiahLujia Atiewin[1], Duo GAO[2], Yun-hang GAO[1*] and Hong-xia MA[1,3*]

[1] Department of Basic Veterinary Medicine, College of Animal Science and Technology, Jilin Agricultural University, Changchun, China.

[2] Liaoning Testing & Inspection Center for Quality & Safety of Veterinary Drugs, Feed and Livestock Products, Liaoning, China.

[3]The Key Laboratory of Animal Production, Product Quality and Security, Ministry of Education, Jilin Agricultural University, Changchun, China.

*Corresponding author:

Hong-Xia MA

College of Animal Science and Technology, Jilin Agricultural University, Xincheng Street No.#2888, Changchun 130118, P.R. China

E-mail: hongxia0731001@163.com

Yun-HangGAO

College of Animal Science and Technology, Jilin Agricultural University, Xincheng Street No.#2888, Changchun 130118, P.R. China

E-mail:gaoyunhang@163.com

¶These authors contributed equally

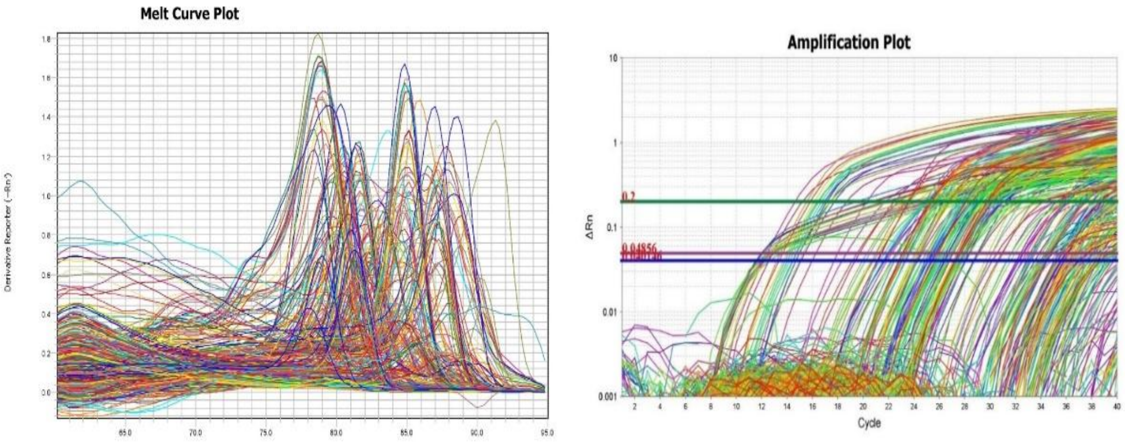

Fig.S1 Amplificationcurve and Meltcurve ofresistance gene

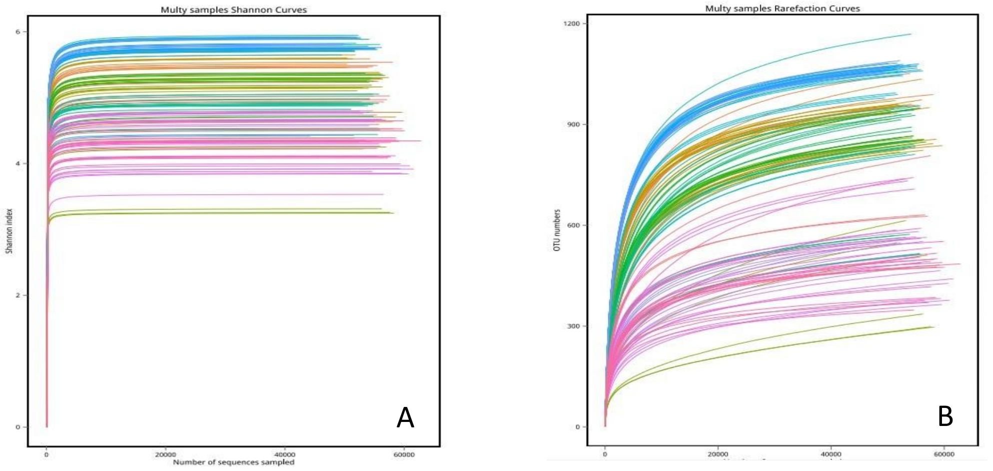

Fig. S2 A: Shannon Indexcurve ; B: Dilution curve

Table S1 Primer sequence of resistance gene

Gene classification	Genes	Sequence（ 5′—3′）	
Aminoglycoside	aacC(6')I 1	GACCGGATTAAGGCCGATG	CTTGCCTTGATATTCAGTTTTTATAACCA
	aacC2	ACGGCATTCTCGATTGCTTT	CCGAGCTTCACGTAAGCATTT
	aacC4	CGGCGTGGGACACGAT	AGGGAACCTTTGCCATCAACT
	strA	CCGGTGGCATTTGAGAAAAA	GTGGCTCAACCTGCGAAAAG
	strB	GCTCGGTCGTGAGAACAATCT	CAATTTCGGTCGCCTGGTAGT
Beta Lactamase	blaSHV	CTTTCCCATGATGAGCACCTTT	TCCTGCTGGCGATAGTGGAT
	blaCTX-M	GGAGGCGTGACGGCTTTT	TTCAGTGCGATCCAGACGAA
	blaTEM	AGCATCTTACGGATGGCATGA	TCCTCCGATCGTTGTCAGAAGT
Tetracycline	tetB	AGTGCGCTTTGGATGCTGTA	AGCCCCAGTAGCTCCTGTGA
	tetC	ACTGGTAAGGTAAACGCCATTGTC	ATGCATAAACCAGCCATTGAGTAAG
	tetE	TTGGCGCTGTATGCAATGAT	CGACGACCTATGCGATCTGA
	tetO	ATGTGGATACTACAACGCATGAGATT	TGCCTCCACATGATATTTTTCCT
	tetQ	CGCCTCAGAAGTAAGTTCATACACTAAG	TCGTTCATGCGGATATTATCAGAAT
MLSB	ermA	TTGAGAAGGGATTTGCGAAAAG	ATATCCATCTCCACCATTAATAGTAAACC
	ermB	TAAAGGGCATTTAACGACGAAACT	TTTATACCTCTGTTTGTTAGGGAATTGAA
	ermC	TTTGAAATCGGCTCAGGAAAA	ATGGTCTATTTCAATGGCAGTTACG
	mphA	CTGACGCGCTCCGTGTT	GGTGGTGCATGGCGATCT
	mphB	CGCAGCGCTTGATCTTGTAG	TTACTGCATCCATACGCTGCTT
Sulfonamide	sul1	CAGCGCTATGCGCTCAAG	ATCCCGCTGCGCTGAGT
	sul2	TCATCTGCCAAACTCGTCGTTA	GTCAAAGAACGCCGCAATGT
Quinolone	qnrA	AGGATTTCTCACGCCAGGATT	CCGCTTTCAATGAAACTGCAA
	qnrS	GTGAGTAATCGTATGTACTTTTGC	AAACACCTCGACTTAAGTCT
Others	intI1	CGAACGAGTGGCGGAGGGTG	TACCCGAGAGCTTGGCACCCA
	MCR-1	CGGTCAGTCCGTTTGTTC	CTTGGTCGGTCTGTA GGG
	NDM-1	ATT AGC CGC TGC ATT GAT	CAT GTC GAG ATAGGA AGT G
	16S rRNA	GGGTTGCGCTCGTTGC	ATGGYTGTCGTCAGCTCGTG

Table S2 The abundance of each drug resistance gene in each group

Types	Different farms				
	BC	CC	CL	YJ	GZL
Aminoglycosides	2.1×10-3	2.9×10-5	1.5×10-3	5.1×10-4	2.4×10-4
β-lactam	5.8×10-5	7.3×10-3	1.6×10-4	1.2×10-3	3.5×10-5
MLSB	2.5×10-3	1.2×10-3	5.5×10-3	9.1×10-3	2.3×10-4
Sulfonamides	1.6×10-3	7.7×10-6	2.8×10-4	5.2×10-4	7.1×10-5
Tetracyclines	9.7×10-3	6.0×10-2	2.8×10-1	2.2×10-1	1.7×10-1
intI1	8.4×10-4	4.1×10-3	2.1×10-3	1.4×10-4	1.9×10-4
MCR-1	1.1×10-7	4.0×10-7	1.4×10-6	2.7×10-7	1.5×10-6
NDM-1	9.0×10-9	4.6×10-8	5.3×10-7	1.1×10-7	4.1×10-8
qnrA	8.2×10-9	0	0	1.9×10-7	2.0×10-8
qnrs	2.1×10-6	2.2×10-7	1.1×10-6	9.8×10-7	2.3×10-7
